# DeepConPred2: An Improved Method for the Prediction of Protein Residue Contacts

**DOI:** 10.1016/j.csbj.2018.10.009

**Published:** 2018-11-10

**Authors:** Wenze Ding, Wenzhi Mao, Di Shao, Wenxuan Zhang, Haipeng Gong

**Affiliations:** a MOE Key Laboratory of Bioinformatics, School of Life Sciences, Tsinghua University, Beijing 100084, China; b Beijing Innovation Center of Structural Biology, Tsinghua University, Beijing 100084, China

**Keywords:** Residue contact prediction, Web server, Protein structure prediction, Contact-assisted folding, Machine learning

## Abstract

Information of residue-residue contacts is essential for understanding the mechanism of protein folding, and has been successfully applied as special topological restraints to simplify the conformational sampling in de novo protein structure prediction. Prediction of protein residue contacts has experienced amazingly rapid progresses recently, with prediction accuracy approaching impressively high levels in the past two years. In this work, we introduce a second version of our residue contact predictor, DeepConPred2, which exhibits substantially improved performance and sufficiently reduced running time after model re-optimization and feature updates. When testing on the CASP12 free modeling targets, our program reaches at least the same level of prediction accuracy as the best contact predictors so far and provides information complementary to other state-of-the-art methods in contact-assisted folding.

## 1 Introduction

Protein residue contact prediction and its corresponding application, contact-assisted protein folding, have become one of the most challenging and promising problems in structural bioinformatics, since the demonstration that native contacts carry sufficient information for the successful reconstruction of protein 3D structures [1]. Indeed, information of residue contacts not only can be integrated into scoring functions to improve the selection of template models [2] or refine the potential function in protein folding simulations [3], but also can be applied as distance constraints to improve the efficiency of conformational sampling or even build tertiary structure models directly [4,5]. As expected, residue contact prediction has become a regular category in recent critical assessment of protein structure prediction (CASP) competitions [6,7]. Particularly, the latest CASP12 competition has reported substantial advance in structure modeling that was mainly driven by the progress in residue contact prediction [8].

Prevalent sequence-based algorithms that predict residue contacts from amino acid sequences can be roughly classified into two categories: methods based on supervised machine learning [[9], [10], [11], [12], [13], [14], [15], [16], [17], [18]] and methods purely based on evolutionary coupling analysis (ECA) [[19], [20], [21], [22], [23], [24]]. Despite the great success, pure ECA-based methods assume that contacting residue pairs should present correlated mutations in the long-term evolution as reflected in the multiple sequence alignment (MSA), but frequently become powerless for targets with limited numbers of homologous sequences [16,20]. In contrast, machine-learning-based methods typically absorb all kinds of information as input features, including the coevolutionary information estimated by ECA-based methods, and thus have become more successful recently. In the past one and half years, a number of methods with similar novel ideas, including RaptorX-Contact, DNCON2 and SPOT-Contact, have been developed, which were reported to achieve the prediction powers that remarkably surpass the best models in the latest CASP12 competition. RaptorX-Contact uses two concatenated deep residual network (ResNet) models to effectively utilize both 1D information like sequence profile as well as predicted secondary structures and 2D information like coevolutionary information as well as pairwise statistical potentials for contact prediction [10]. DNCON2 is made up of several deep convolutional neural network (CNN) blocks, where five CNN blocks are trained to produce preliminary predictions at different thresholds of residue distances and one additional CNN block is used to combine these preliminary results to produce the final contact prediction [11]. SPOT-Contact adopts a deep hybrid network: prepared inputs are fed into a ResNet model, and the outputs are further processed by a 2D-Bidirectional-ResLSTM model [18].

Our previous contact predictor DeepConPred [17] could reach comparable performance to top methods in the CASP12 competition. In this work, we introduce a second version of this predictor, DeepConPred2. After re-optimizing the model architecture and updating features, the new version exhibits substantially improved performance (e.g., prediction accuracy for the top *L*/5 predicted contacts rises from 44.0% to 69.6% for the CASP12 targets), and reaches a level comparable to the other state-of-the-art methods including RaptorX-Contact, DNCON2 and SPOT-Contact. On the other hand, the new version was implemented with GPU acceleration, and thus the running time has been sufficiently reduced, especially for large protein targets. Finally, we developed a user friendly online server of DeepConPred2 to provide service for the prediction of residue contact maps.

## 2 Methods

**Fig. 1:**
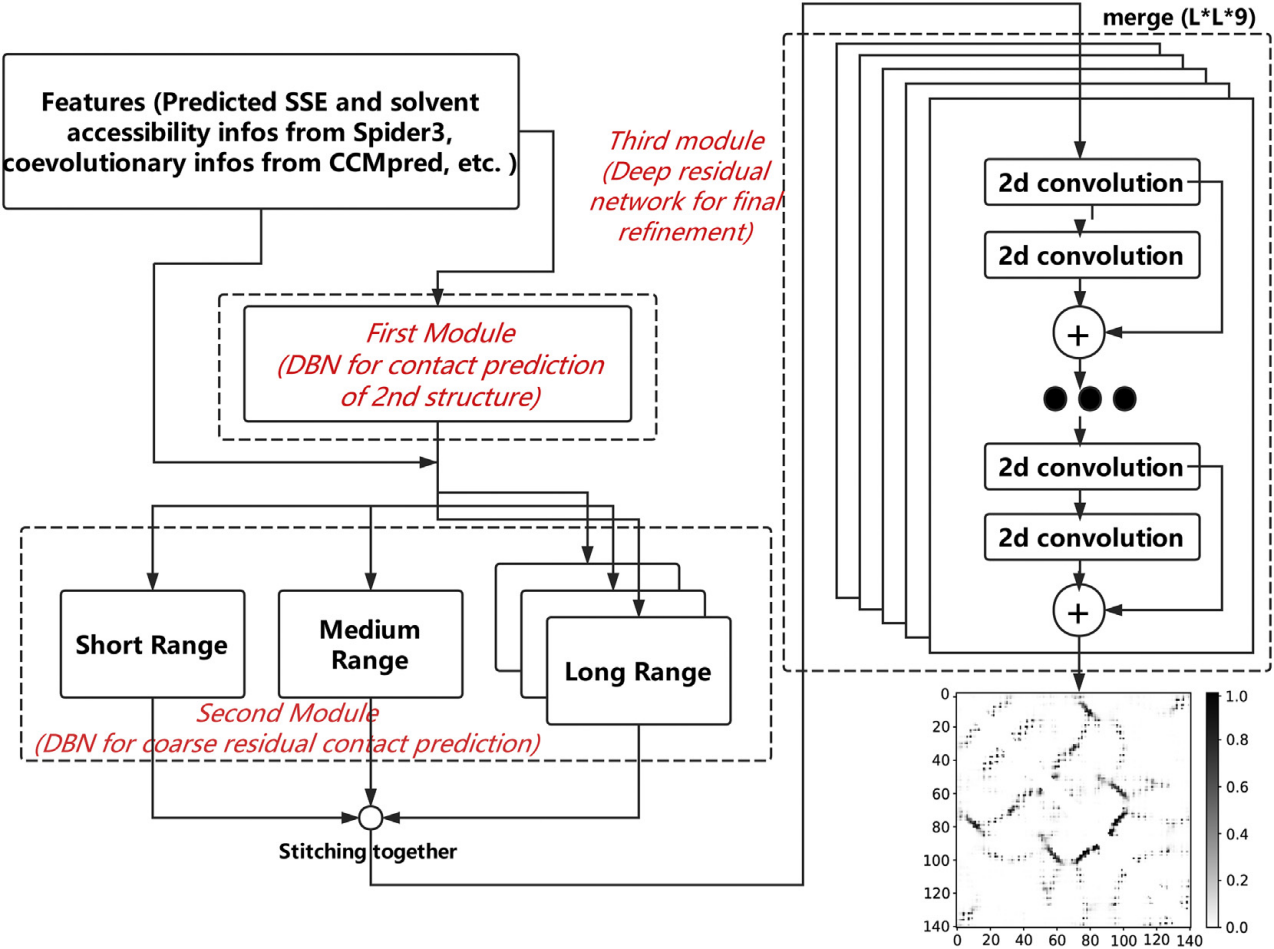
The schematic layout of DeepConPred2. Three modules are framed with dashed lines and marked with red italic.

The flow chart of DeepConPred2 is shown in Fig. 1. Similar to the previous version, DeepConPred2 could be divided to three stages/modules. In the first module, a deep belief network (DBN) model was adopted to predict contacts between secondary structure elements (SSEs). In the second module, results of the first module as well as other features were fed to DBN models to predict the residue contacts. The previous version only focused on contacts between long-range (sequence separation ≥ 24) residue pairs. Here, in this new version, we developed additional DBN models to process the medium- (12 ≤ sequence separation ≤ 23) and short-range (6 ≤ sequence separation ≤ 11) residue pairs. In the third module, prediction results of all three categories of contacts from the second module as well as the coevolutionary information were combined to further refine the predicted residue contact map. Particularly, in the new version, we substituted the DBN model with the ResNet [25,26] model that has shown great successes in contact prediction.

### 2.1 Datasets

We merged the training set and test set used in the study by Xiong et al. [17] as our new training set. Specifically, all proteins in the training set of this work came from the SCOPe 2.05 version [27]. After redundancy elimination with a cutoff of 20% sequence identity, only one protein was retained from each superfamily to guarantee the correct representation of fold topology. Finally, our training set contained 3443 protein domains.

We prepared an independent test set of 77 protein domains that belonged to novel superfamilies. Specifically, we extracted protein domains newly released in the SCOPe 2.07 version [28] (in comparison to the SCOPe 2.05 version) and kept domains that were longer than 50 residues and did not contain multiple structures or missing backbone atoms. After removing redundancy from the training set with a cutoff of 20% sequence identity, we extracted the shortest protein domain from each new superfamily to compose the test set.

Besides the independent test set, we adopted the CASP11 and CASP12 protein sets as additional testing sets to objectively evaluate the performance of our new model against the previous version. When comparing our model with other state-of-the-art methods, we mainly made the evaluation on 22 CASP12 free modeling (FM) targets, the hardest targets in de novo protein structure prediction, and then extended the evaluation to all CASP12 targets, including additional 31 template-based modeling (TBM) ones.

### 2.2 The First Module

We updated some input features in the first module. Specifically, we adopted Spider3 [29] secondary structure predictions to replace the SSpro [30] results used previously, and adopted CCMpred [19] to calculate the coevolutionary information instead of the previous plmDCA [21]. The overall architecture of the DBN model was changed from the previous hourglass shape to a more balanced 133–400–400–400–3, where 133 is the dimension of input feature vectors and 3 is the output dimension including the corresponding probabilities of parallel contact, anti-parallel contact and non-contact for each SSE pair.

### 2.3 The Second Module

Our previous version only processes long-range residue pairs. In the new version, we developed two additional DBN models to predict the contacts between short- and medium-range residue pairs. The short-, medium- and long-range models read in very similar features. However, we updated some features such as coevolutionary information (from CCMpred instead of plmDCA), predicted secondary structure and solvent accessibility (both from Spider3 instead of the previous SSpro and ACCpro [30]), and some statistical information. The overall architecture of all DNB models was adjusted to d-800-700-600-2, where d is the dimension of input feature vectors and was set to 416, 480 and 478 for short-, medium- and long-range models, respectively. We adopted all samples for the training of short- and medium-range DBN models. For the long-range prediction, however, the total amount of samples was extremely huge, mainly dominated by negative ones. So we retained all positive samples but down-sampled the negative ones to keep their ratio to roughly 1:1. To avoid overtraining, we adjusted the weights for positive and negative samples in the loss function to keep fidelity of its origin proportion in model training. The original positive-negative ratio of our training set is around 1:50. In practice, we developed three models with relative weights of 1:40, 1:50 and 1:60 in the loss function. The average of these three models were then taken as the results of long-range prediction in the second module.

### 2.4 The Third Module

In the third module, we optimized five ResNet models with slight variation of architecture and finally took the ensemble average of these models as the final prediction (Table 1). The input features of each ResNet model include outcomes of the second module, position of the target residue pair in the overall contact map, secondary structural information predicted from Spider3 and coevolutionary information provided by CCMpred. During the training of each ResNet model, we treated all samples in each individual protein as a mini-batch and thus the input pairwise features had the dimension of *L* × *L* × 9, where *L* is the length of the protein and 9 can be interpreted as the number of features for one pixel on the contact map (i.e. the target residue pair). We adopted 64 3 × 3 2D convolution filters for each convolutional layer. The convolution stride was set as 1 and the zero-padding pattern was set as ‘same’ to make feature maps of all layers have the same shape as the original input (i.e. *L* × *L*). Leaky rectified linear unit (leaky-ReLU) activation function was used for non-linear transformation. The depth of our ResNet models varied from 50 to 80. By stacking many residual blocks, our networks could capture interdependency of residue pairs of very long range even though we used a small filter size.

**Table 1:** ResNet models in the third module.

	Number of layers	Activation	F1-score
Model 1	50	Pre-activation	0.5419
Model 2	60	Pre-activation	0.5527
Model 3	70	Pre-activation	0.5495
Model 4	80	Pre-activation	0.5561
Model 5	80	Post-activation	0.5508
Ensemble	Average of above 5 models		0.5711

Due to the limitation of memory usage when training the ResNet models, length of training input proteins was limited to no >400. For each of the 128 protein domains that exceed this limit, we randomly chose 4 overlapping 400 × 400 subplots as input.

### 2.5 Evaluation

All parameters were optimized using 5-fold cross validation on the training set. In this process, we adopted the F1-score for all available residue pairs to evaluate individual models. The F1-score is defined as the harmonic mean of Precision and Recall:

(1) $$F1‐\text{score}=\frac{2\times \text{Precision}\times \text{Recall}}{\text{Precision}+\text{Recall}}$$

where Precision and Recall evaluate the proportions of true positives within all positive predictions and within all true samples, respectively:

(2) Precision=TruePositivesTruePositives+FalsePositivesRecall=TruePositivesTruePositives+FalseNegatives

After model training, performance of the algorithm was then independently evaluated on three testing sets: the independent test set, CASP11 set and CASP12 set. According to standard CASP definition [31], residues with Euclidean distance between two C_β_ atoms falling within 8.0 Å were considered to be in contact. Following the CASP routine, we chose the precisions (Eq. 2) for the top *L*/10, *L*/5, *L*/2 and *L* predicted residue pairs as the main evaluators, where *L* is the length of the protein.

In this work, we compared our method with three state-of-the-art algorithms, DNCON2, RaptorX-Contact and SPOT-Contact. The comparison was first performed on 22 CASP12 FM targets that have available structure information. Prediction results of DNCON2 (http://sysbio.rnet.missouri.edu/dncon2/), RaptorX-Contact (http://raptorx.uchicago.edu/ContactMap/) and SPOT-Contact (http://sparks-lab.org/jack/server/SPOT-Contact/) were obtained from their web servers in July 2018, respectively. Then, we extended the comparison to the overall 53 CASP12 targets that have available structure information, including additional 31 TBM ones. Results of additional targets were received on October 5th, 2018 from the online servers of RaptorX-Contact and SPOT-Contact. The server of DNCON2 was unavailable for new predictions at that time for some reasons. Thus, we had to use the latest downloadable results of the CASP12 test posted on the website, which only contained 35 targets.

To evaluate the contribution of contact predictors in protein structure prediction, we modeled the structures of the 22 CASP12 FM protein targets (and then extended to all available CASP12 targets) using the top 2*L* contacts predicted by DeepConPred2, DNCON2, RaptorX-Contact and SPOT-Contact, respectively. Here, we adopted the CONFOLD program [4] to proceed the ab initio folding without using any additional force fields. Specifically, the top 2*L* predicted residue pairs were restrained between 3.5 and 8 Å, whereas all other parameters were taken as the default values. The best model (with the lowest root-mean-square-distance (RMSD) to the native structure) among the top 5 ones as reported by CONFOLD was taken as the representative structural model of each target for various contact predictors.

## 3 Results and Discussion

### 3.1 Training and Cross Validation

In the first and second modules, we only retained the best DBN models based on F1-scores in the 5-fold cross validation. In the third module, however, we constructed ResNet models with various levels of depths. In the final prediction, ensemble average of these models was adopted to further improve the performance (Table 1).

Average ensemble is also used in RaptorX-Contact and SPOT-Contact [10,18], which allows predictors to reduce the influence of generalization on their own training sets. Because of the variation of architectures, parameters initializations, data feeding and other factors, each network in the ensemble captures a slightly different pattern. Ensemble averaging makes use of the complementarity of these patterns and thus could improve the performance. The following equation provides illustration:

(3) G=1T∑tgtEErrg=1T∑tErrgt=1T∑tgt−f2=1T∑tgt−G2+G−f2≥G−f2=ErrG

where E, g_t_, G, f and Err represent expectation, the t-th model in the ensemble, ensemble average, the truth ground (label) and model error, respectively. In this work, F1-score of the ensemble (0.5711) surpasses all individual models.

### 3.2 Evaluation in the Testing Sets

We evaluated the performance of DeepConPred2 over the previous version on three testing sets. The previous version can only predict contacts of long-range residue pairs while the new version has no such limits. As shown in Table 2, Table 3, Table 4, the new version performs markedly better than the previous one on all three testing sets, regardless of the amount of top predicted contacts that are chosen for evaluation. For example, the precision of top *L*/5 predicted long-range residue pairs reaches 40.61%, 43.35% and 44.00% on the independent test set, CASP11 set and CASP12 set respectively for the old version, but rises to 70.67%, 71.26% and 69.60% respectively in the new version. Balanced performance of our new program on the three testing sets also supports robustness of this method for different kinds of targets.

**Table 2:** Performance evaluation on the independent test set.

Range	Version	*L*/10	*L*/5	*L*/2	*L*
Short	New	0.7539	0.6667	0.5134	0.3622
	Old	–	–	–	–
Medium	New	0.6926	0.6176	0.4871	0.3806
	Old	–	–	–	–
Long	New	0.7411	0.7067	0.6294	0.5378
	Old	0.5517	0.4061	0.3414	0.2993

**Table 3:** Performance evaluation on the CASP11 set.

Range	Version	*L*/10	*L*/5	*L*/2	*L*
Short	New	0.8345	0.7479	0.5680	0.3978
	Old	–	–	–	–
Medium	New	0.7881	0.7314	0.6135	0.4617
	Old	–	–	–	–
Long	New	0.7315	0.7126	0.6619	0.5695
	Old	0.5306	0.4335	0.3813	0.2935

**Table 4:** Performance evaluation on the CASP12 set.

Range	Version	*L*/10	*L*/5	*L*/2	*L*
Short	New	0.7075	0.6689	0.5152	0.3600
	Old	–	–	–	–
Medium	New	0.6966	0.6568	0.5211	0.3860
	Old	–	–	–	–
Long	New	0.7039	0.6960	0.6225	0.5310
	Old	0.5157	0.4400	0.3138	0.2579

Generally, in comparison to the old version, the new version mainly includes modifications on three facets: A) updates of input features and architecture fine-tuning of the DBN models in the first and second module; B) consideration of short- and medium-range predictions in the second module; and C) adoption of ResNet in the third module. We roughly estimated their contributions to the performance improvement for long-range residue contacts on the independent test set. As shown in Table S1, modification A brings the largest improvement in prediction accuracy (by ~16 percentage points), followed by modification C, which contributes by ~10 percentage points, while modification B only makes a minor contribution.

**Fig. 2:**
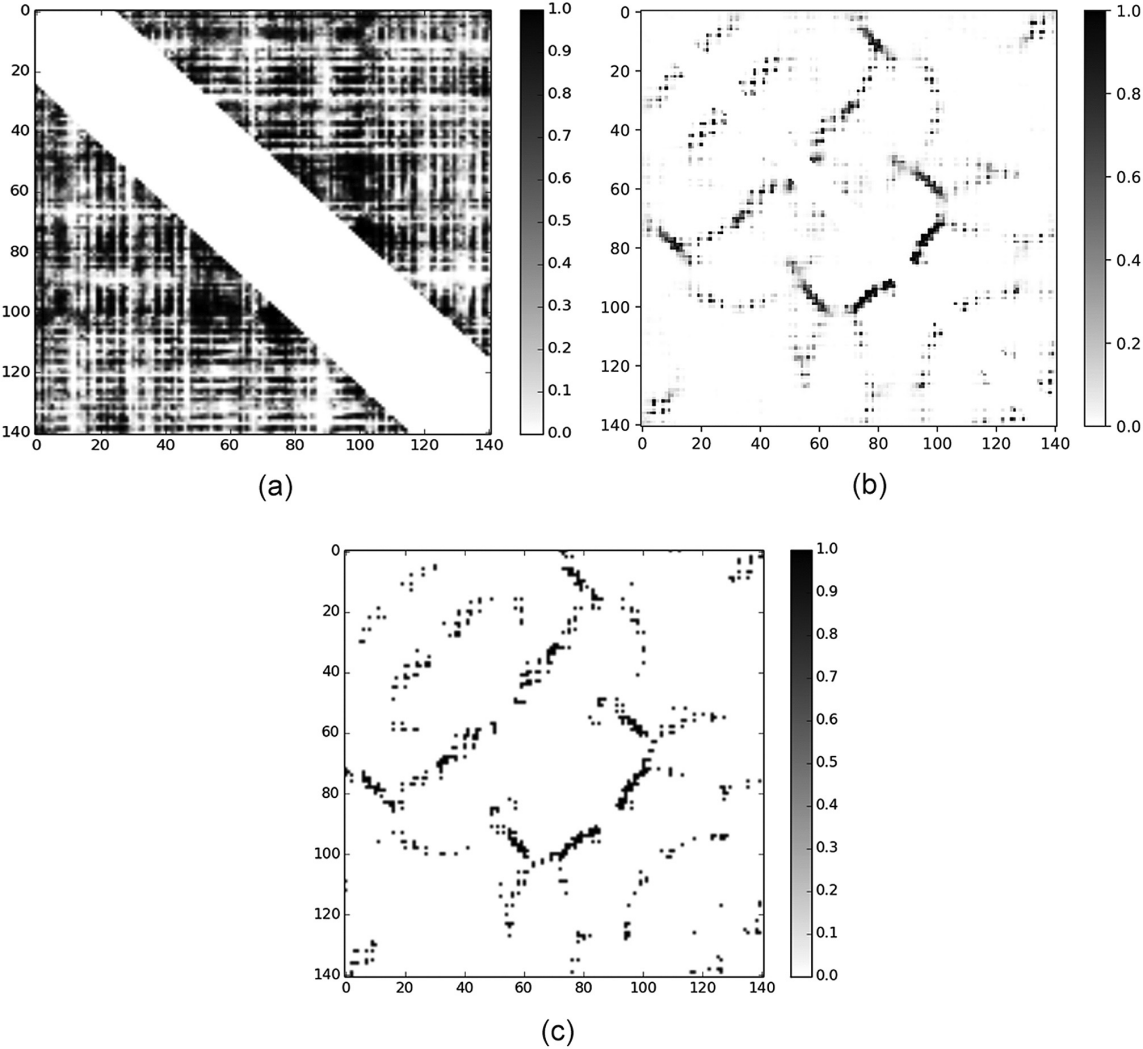
Comparison of contact maps for a specific target (PDB ID: 1A3A). (a) The contact map predicted by our previous version. (b) The contact map predicted by the new version. (c) The contact map of the native structure.

Moreover, the new version greatly reduces the noise levels in the predicted residue contact maps. For an example target, the previous version generates a contact map with serious palisade noises that cover useful contact information (Fig. 2a). In our new version, palisade noises are effectively removed by the use of weighted loss function and ensemble trick in the second module (Fig. S1) and predicted values are rescaled to fit its real probability through the ultra-deep ResNet in the third module (Fig. 2b), which jointly make the outcome closer to its native form (Fig. 2c).

**Fig. 3:**
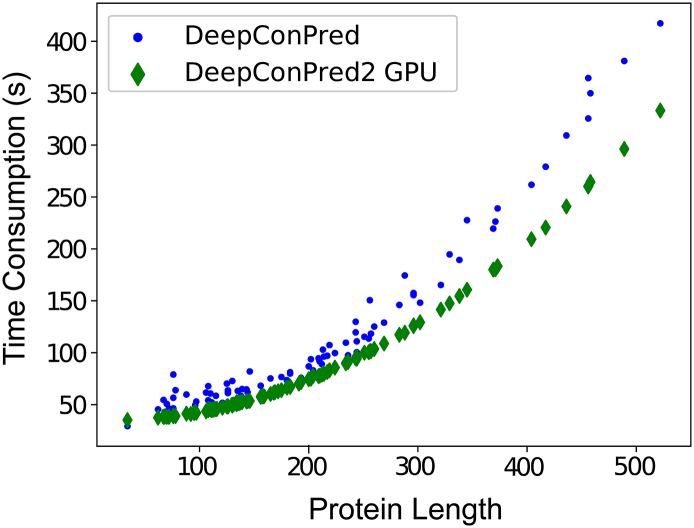
Consumption of computational time of different versions on 102 CASP11 targets.

In addition to the substantial improvement in prediction accuracy and map quality, the new version was implemented using the TensorFlow framework [32] with GPU acceleration. We also tested the running time of the new version over the previous one on 101 CASP11 protein targets. Despite the extra computational expenses for the additional models (e.g., short- and medium-range DBN models and multiple models for ensemble averaging), the new version still runs faster than the previous one, especially for large proteins (Fig. 3). For example, at target length of 456, our new version takes 104 s less than the old one, which corresponds to a speed-up by 28.6% (from 364.6 s to 260.3 s).

To provide convenient service for residue contact prediction, we also constructed a web server of DeepConPred2. Users could submit the protein sequence on the website and approach the prediction results later by email notification. Meanwhile, program for local execution is also provided for free download.

### 3.3 Comparison With Other Cutting-Edge Methods

We first compare our method with three state-of-the-art algorithms, DNCON2, RaptorX-Contact and SPOT-Contact, on 22 CASP12 FM targets. As shown in Table 5, DeepConPred2 outperforms DNCON2 in all categories (e.g., long-range top *L*/5 precision: 57.56% vs. 53.49%). When compared with RaptorX-Contact, DeepConPred2 shows similar performance for long-range contacts. For example, the long-range top *L*, *L*/2 and *L*/5 precisions of DeepConPred2 vs. RaptorX-Contact are 41.27% vs. 39.50%, 49.16% vs. 51.15% and 57.56% vs. 58.55%, respectively. As for short- and medium-range contacts, DeepConPred2 exhibits an advantage to RaptorX-Contact. For medium-range contacts, the top *L*, *L*/2 and *L*/5 precisions of DeepConPred2 vs. RaptorX-Contact are 30.78% vs. 23.64%, 42.63% vs. 36.08% and 56.87% vs. 53.41%, respectively, while for short-range contacts, they are 31.90% vs. 22.57%, 44.34% vs. 35.83% and 58.49% vs. 57.20%, respectively. DeepConPred2 is slightly prevailed by SPOT-Contact (e.g., the long-range top *L*, *L*/2 and *L*/5 precisions of DeepConPred2 vs. SPOT-Contact are 41.27% vs. 43.93%, 49.16% vs. 52.69% and 57.56% vs. 63.18%, respectively).

**Table 5:** Comparison of prediction precisions on 22 CASP12 FM targets.

Range	Methods	*L*/10	*L*/5	*L*/2	*L*
Short	DNCON2	0.5219	0.5145	0.3879	0.2807
	RaptorX-Contact	0.6871	0.5720	0.3583	0.2257
	SPOT-Contact	0.7220	0.6146	0.3994	0.2421
	DeepConPred2	0.6257	0.5849	0.4434	0.3190
Medium	DNCON2	0.4698	0.4682	0.3837	0.2859
	RaptorX-Contact	0.6104	0.5341	0.3608	0.2364
	SPOT-Contact	0.7120	0.6195	0.4182	0.2699
	DeepConPred2	0.6091	0.5687	0.4263	0.3078
Long	DNCON2	0.5864	0.5349	0.4241	0.3378
	RaptorX-Contact	0.6765	0.5855	0.5115	0.3950
	SPOT-Contact	0.6758	0.6318	0.5269	0.4393
	DeepConPred2	0.6100	0.5756	0.4916	0.4127

We then extended the comparison to all 53 CASP12 targets that have available structure information (Tables S2–S3). Because DNCON2 server was temporarily unavailable for prediction, comparison with DNCON2 was only conducted on 35 targets that have downloadable prediction results posted on the DNCON2 website. Similar to the previous comparison, DeepConPred2 significantly outperforms DNCON2, but is slightly prevailed by SPOT-Contact. Possibly due to the strong prediction power on TBM targets, the relatively easy targets for de novo protein structure prediction, performance of RaptorX-Contact slightly surpasses DeepConPred2 and becomes close to SPOT-Contact. Nevertheless, DeepConPred2 reaches a high level of prediction accuracy (especially for the hard FM targets) and thus can be ranked as one of the best residue contact predictors. It should be noticed that updates of sequence databases, especially the introduction of metagenome sequence data, would have big impact on performance of predictors [33]. It is possible that the performance of our method would be further improved though database updating, especially for TBM targets that potentially will have more homologous sequences identified from the metagenome data.

### 3.4 Evaluation for Contact-Assisted Folding

To further validate the contribution of predicted contacts by various methods in the practical protein structure prediction, we first constructed the model structures for the 22 CASP12 FM targets with the general CONFOLD protocol, using the top 2*L* predicted contacts as the only constraints. For each protein, we chose the one with minimum RMSD to the native structure from the top 5 models provided by CONFOLD as its representative model for the corresponding algorithm. We performed Levene test to make sure the equal variance between RMSD groups of different methods and then conducted 2-sided paired *t*-test to check whether there are any significant differences. At the confidence threshold of 0.1, CONFOLD models using contact prediction of our method surpass those using DNCON2 significantly but have no significant differences from those of RaptorX-Contact or SPOT-Contact (Table 6).

**Table 6:** *P*-values of paired t-test and Levene-test on CONFOLD RMSD groups from different methods.

Paired t-test/Levene test	DNCON2 (12.50)	RaptorX-Contact (11.82)	SPOT-Contact (10.43)
DeepConPred2 (11.60)	0.07238/0.9463	0.8031/0.6597	0.1145/0.8913

P-values of the paired t-test and Levene test are listed before and after the slash, respectively. Numbers in brackets are the average RMSD values of the 22 CASP12 FM targets for corresponding algorithms.

**Fig. 4:**
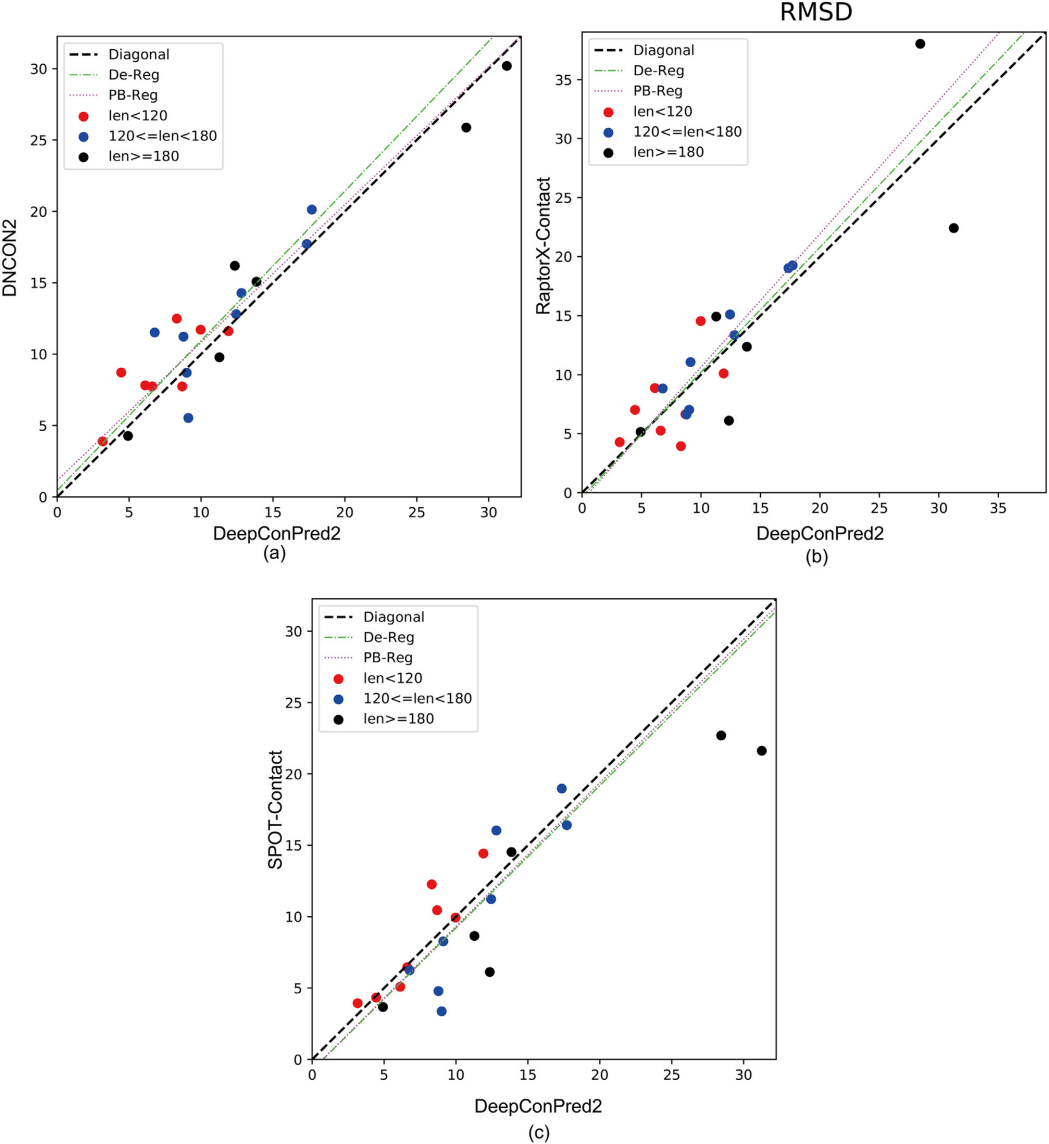
RMSD comparison between CONFOLD results of models generated using our program and other 3 methods on 22 CASP12 FM targets. Each point denotes a protein target, with various colors labeling the proteins of different sizes: red for small domains (length <120), blue for medium domains (120 ≤ length < 180), and black for large domains (length ≥ 180). The lime green dashed lines and fuchsia dotted lines denote the results of Deming regression and the Passing-Bablock regression, respectively.

Fig. 4 shows the pairwise comparison between models generated using our method vs. those using the other three methods by the RMSD criterion. Clearly, in respect of contact-assisted folding, DeepConPred2 outperforms DNCON2 and reaches a very similar power to RaptorX-Contact. Although slightly prevailed by SPOT-Contact, DeepConPred2 could provide complementary information: our method outperforms SPOT-Contact on small proteins (red points in Fig. 4c) but performs less well on large proteins (black points in Fig. 4c), which might arise from the differences in average protein lengths between the training sets of the two methods. Comparisons using TM-score and GDT-TS score show the similar trend (Figs. S2–S3). For a comprehensive and systematic comparison over the models produced using these methods, we also performed Deming regression and Passing-Bablock regression analyses (Tables S4–S6). Interestingly, these two statistics tests deny the presence of significant differences between the four methods.

We then extend our folding test to all available CASP12 targets. As shown in Fig. S4, despite the generally comparable model quality produced by the four methods, information complementarity is present between these methods (see the dispersion of points). Deming regression and Passing-Bablock regression analyses (Tables S7–S9) also indicate that the four methods have the same level of powers on contact-assisted folding.

Despite the CASP12 report that usage of coevolutionary information and ultra-deep learning framework has raised accuracy performance of contact prediction into a new level [8], our analysis shows that apparent gaps of performance between different state-of-the-art contact predictors are not linearly reflected in their real contributions to the practical protein structure prediction, at least in the contact-assisted folding using CONFOLD. Although this phenomenon should be validated using other folding programs, we can still suspect that state-of-the-art contact predictors make a similar level of contribution to tertiary structure modeling as long as the accuracy of contact prediction exceeds some threshold. Thus, to break through the bottleneck, effectively utilizing the predicted contact information, for instance, combining complementary results of various state-of-the-arts contact predictors, may further benefit practical protein structure prediction.

## 4 Conclusions

In this work, we present a second version of our residue contact predictor, DeepConPred2, which can predict all contacts of a protein at once from a protein sequence. The new version exhibits remarkably improved performance in comparison to the previous one, and could reach a prediction power comparable to the other state-of-the-art methods. In addition, implementation with GPU acceleration further reduces the running time, especially for large protein targets. A user friendly web server is available for reliable prediction of residue contact maps.

## Appendix A Supplementary data

Supplementary material Image 1
