# Supplementary material for: DeepConPred2: An Improved Method for the Prediction of Protein Residue Contacts
Source: Comput Struct Biotechnol J. 2018 Nov 10;16:503–10. doi: 10.1016/j.csbj.2018.10.009 (PMC6247404; doi:10.1016/j.csbj.2018.10.009)
Supplement: Supplementary file 1 — Supplementary material [file mmc1.pdf]

Supplementary figures

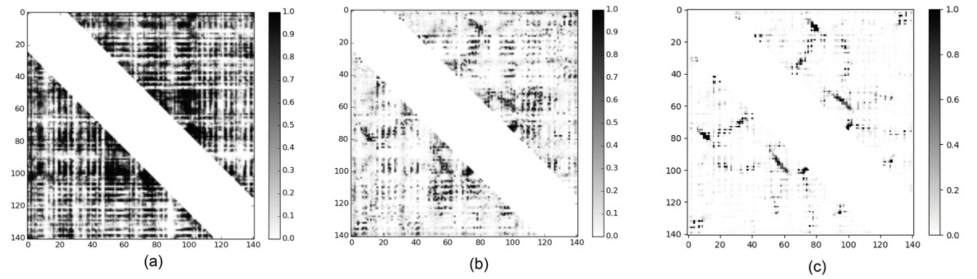

Figure S1. Demonstration for the effect on removal of palisade noises by use of weighted loss and ensemble trick in the second module (PDB ID: 1A3A). **(a)** The old version without readjustment of weights in the loss function. **(b)** A relative weight of 1:20 applied for positive to negative in the loss function. **(c)** Ensemble average of 3 models with weighted loss of 1:40, 1:50 and 1:60.

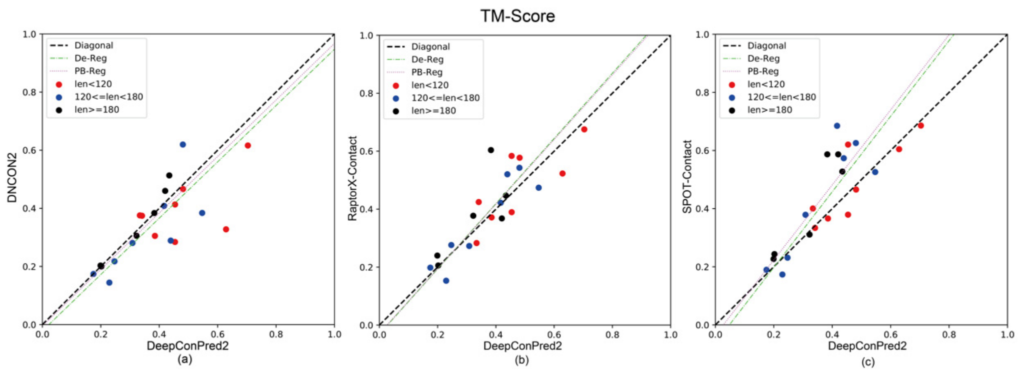

Figure S2. TM-Score comparison between CONFOLD results of models generated using our program and other 3 methods on 22 CASP12 FM targets. Each point denotes a protein target, with various colors labeling the proteins of different sizes: red for small domains (length <120), blue for medium domains (120 ≤ length < 180), and black for large domains (length ≥ 180). The lime green dashed lines are the results of Deming regression and the fuchsia dotted lines are the results of Passing-Bablock regression.

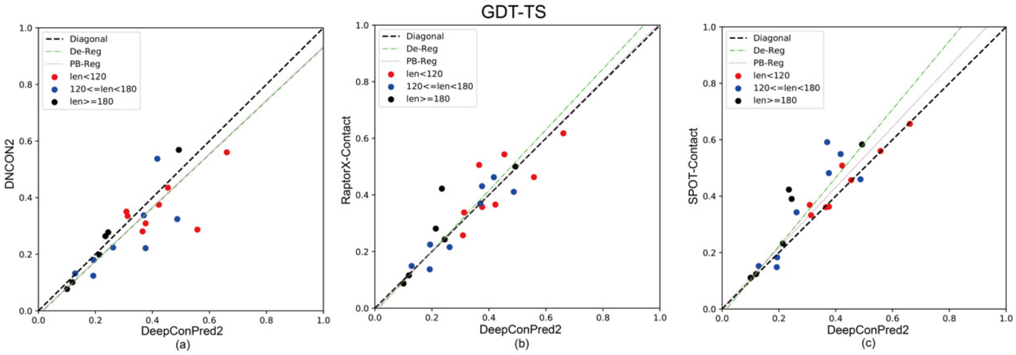

Figure S3. GDT-TS comparison between CONFOLD results of models generated using our program and other 3 methods on 22 CASP12 FM targets. Each point denotes a protein target, with various colors labeling the proteins of different sizes: red for small domains (length <120), blue for medium domains (120 $\leq$ length < 180), and black for large domains (length $\geq$ 180). The lime green dashed lines are the results of Deming regression and the fuchsia dotted lines are the results of Passing-Bablock regression.

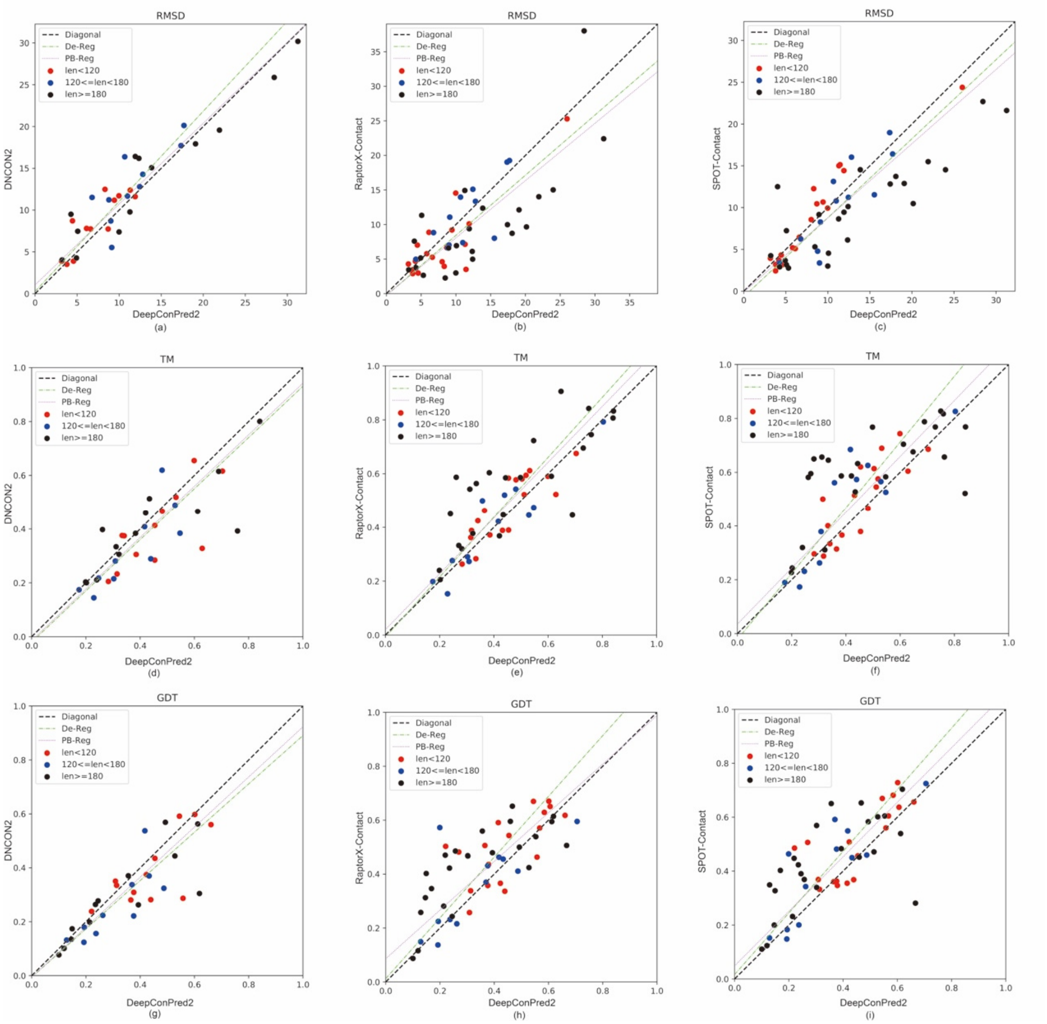

Figure S4. RMSD, TM-Score and GDT-TS comparisons between CONFOLD results of models generated using our program and other 3 methods on all 53 CASP12 targets. Each point denotes a protein target, with various colors labeling the proteins of different sizes: red for small domains (length <120), blue for medium domains (120 ≤ length < 180), and black for large domains (length ≥ 180). The lime green dashed lines are the results of Deming regression and the fuchsia dotted lines are the results of Passing-Bablock regression.

Supplementary tables

Table S1. Step-by-step performance improvement of long-range predictions on the independent test set.

Stepwise model changes	L/5	L/2	L
**Original DeepConPred**	0.4061	0.3414	0.2993
**Modification A**	0.5681	0.5065	0.4429
**Modifications A+B**	0.5812	0.5183	0.4471
**Modifications A+B+C (i.e. DeepConPred2)**	0.7067	0.6294	0.5378

DeepConpred2 contains the following changes: A) updates of input features and architecture fine-tuning of the DBN models in the first and second module; B) consideration of short- and medium-range predictions in the second module; and C) adoption of ResNet in the third module.

Table S2. Comparison of prediction accuracies on all 53 CASP12 targets by DeepConPred2, RaptorX-Contact and SPOT-Contact.

Range	Methods	L/10	L/5	L/2	L
**Short**	RaptorX-Contact	0.7601	0.6667	0.4293	0.2637
	SPOT-Contact	0.7711	0.6886	0.4508	0.2756
	DeepConPred2	0.7075	0.6689	0.5152	0.3600
**Medium**	RaptorX-Contact	0.7442	0.6833	0.4730	0.3048
	SPOT-Contact	0.7747	0.6941	0.5030	0.3189
	DeepConPred2	0.6966	0.6568	0.5211	0.3860
**Long**	RaptorX-Contact	0.8091	0.7268	0.6532	0.5323
	SPOT-Contact	0.7945	0.7412	0.6553	0.5315
	DeepConPred2	0.7039	0.6960	0.6225	0.5310

Table S3. Comparison of prediction accuracies on 35 CASP12 targets with available DNCON2 results by DeepConPred2 and DNCON2.

Range	Methods	L/10	L/5	L/2	L
**Short**	DNCON2	0.5305	0.5244	0.4028	0.2975
	DeepConPred2	0.6592	0.6391	0.4846	0.3395
**Medium**	DNCON2	0.5364	0.5282	0.4277	0.3193
	DeepConPred2	0.6513	0.6165	0.4734	0.3501
**Long**	DNCON2	0.6010	0.5496	0.5005	0.4254
	DeepConPred2	0.6393	0.6308	0.5396	0.4563

Table S4. Deming regression and Passing-Bablock regression analyses for the RMSD comparison between CONFOLD results of models generated using our program and other 3 methods on 22 CASP12 FM targets.

Method Pairs	Regression	Parameters	Estimated Values	Lower bound *	Upper bound *
**DNCON2 vs. DeepConPred2**	Deming	Slope	1.048	0.8920	1.205
		Intercept	0.4538	-1.151	2.059
	Passing-Bablock	Slope	0.9655	0.8128	1.210
		Intercept	1.160	-1.491	3.221
**RaptorX-Contact vs. DeepConPred2**	Deming	Slope	1.057	0.6839	1.430
		Intercept	-0.3495	-3.631	2.932
	Passing-Bablock	Slope	1.130	0.7558	1.501
		Intercept	-0.6599	-5.263	2.773
**SPOT-Contact vs. DeepConPred2**	Deming	Slope	0.9965	0.6605	1.332
		Intercept	-0.7484	-3.727	2.231
	Passing-Bablock	Slope	1.006	0.7652	1.400
		Intercept	-0.7370	-4.261	1.349

*with confidence of 95%.

Table S5. Deming regression and Passing-Bablock regression analyses for TM-Score comparison between CONFOLD results of models generated using our program and other 3 methods on 22 CASP12 FM targets.

**Method Pairs**	**Regression**	**Parameters**	**Estimated Values**	**Lower bound ***	**Upper bound ***
**DNCON2 vs. DeepConPred2**	Deming	Intercept	-0.0215	-0.1207	0.0777
		Slope	0.9690	0.6430	1.2951
	Passing-Bablock	Intercept	-0.0047	-0.1314	0.0814
		Slope	0.9719	0.6712	1.3411
**RaptorX-Contact vs. DeepConPred2**	Deming	Intercept	-0.0315	-0.1405	0.0776
		Slope	1.1257	0.8029	1.4484
	Passing-Bablock	Intercept	-0.0288	-0.1846	0.0712
		Slope	1.1153	0.8377	1.5010
**SPOT-Contact vs. DeepConPred2**	Deming	Intercept	-0.0622	-0.1646	0.0402
		Slope	1.3001	0.9574	1.6428
	Passing-Bablock	Intercept	-0.0349	-0.2635	0.0289
		Slope	1.2912	0.9582	1.9049

*with confidence of 95%.

Table S6. Deming regression and Passing-Bablock regression analyses for GDT-TS comparison between CONFOLD results of models generated using our program and other 3 methods on 22 CASP12 FM targets.

Method Pairs	Regression	Parameters	Estimated Values	Lower bound *	Upper bound *
**DNCON2 vs. DeepConPred2**	Deming	Intercept	-0.0145	-0.0450	0.0160
		Slope	0.9444	0.7585	1.1303
	Passing-Bablock	Intercept	-0.0121	-0.0989	0.0401
		Slope	0.9455	0.7295	1.3027
**RaptorX-Contact vs. DeepConPred2**	Deming	Intercept	-0.0144	-0.0448	0.0160
		Slope	1.0750	0.9152	1.2349
	Passing-Bablock	Intercept	-0.0052	-0.0692	0.0569
		Slope	1.0140	0.8214	1.2744
**SPOT-Contact vs. DeepConPred2**	Deming	Intercept	-0.0186	-0.0523	0.0151
		Slope	1.2103	1.0282	1.3923
	Passing-Bablock	Intercept	0.0033	-0.0740	0.0411
		Slope	1.0697	0.9305	1.3956

*with confidence of 95%.

Table S7. Deming regression and Passing-Bablock regression analyses for RMSD comparison between CONFOLD results of models generated using our program and other 3 methods on all available CASP12 targets.

Method Pairs	Regression	Parameters	Estimated Values	Lower bound *	Upper bound *
**DNCON2 vs. DeepConPred2**	Deming	Intercept	0.2742	-0.8378	1.3862
		Slope	1.0776	0.9385	1.2166
	Passing-Bablock	Intercept	1.0501	-0.6145	2.6808
		Slope	0.9671	0.8383	1.1489
**RaptorX-Contact vs. DeepConPred2**	Deming	Intercept	-0.2670	-1.6922	1.1582
		Slope	0.8702	0.6399	1.1005
	Passing-Bablock	Intercept	-0.2182	-2.9483	1.4242
		Slope	0.8287	0.6218	1.1074
**SPOT-Contact vs. DeepConPred2**	Deming	Intercept	-0.6606	-1.7225	0.4012
		Slope	0.9451	0.7835	1.1067
	Passing-Bablock	Intercept	-0.0869	-1.7249	1.0594
		Slope	0.8868	0.7218	1.0971

*with confidence of 95%.

Table S8. Deming regression and Passing-Bablock regression analyses for TM-Score comparison between CONFOLD results of models generated using our program and other 3 methods on all available CASP12 targets.

Method Pairs	Regression	Parameters	Estimated Values	Lower bound *	Upper bound *
**DNCON2 vs. DeepConPred2**	Deming	Intercept	-0.0199	-0.0863	0.0465
		Slope	0.9506	0.7388	1.1623
	Passing-Bablock	Intercept	-0.0142	-0.0934	0.0641
		Slope	0.9564	0.7490	1.1625
**RaptorX-Contact vs. DeepConPred2**	Deming	Intercept	-0.0077	-0.0816	0.0662
		Slope	1.1141	0.9195	1.3087
	Passing-Bablock	Intercept	0.0201	-0.0331	0.0782
		Slope	1.0371	0.8787	1.1975
**SPOT-Contact vs. DeepConPred2**	Deming	Intercept	-0.0212	-0.0894	0.0469
		Slope	1.2224	1.0233	1.4214
	Passing-Bablock	Intercept	0.0354	-0.0619	0.1273
		Slope	1.0388	0.8551	1.2561

*with confidence of 95%.

Table S9. Deming regression and Passing-Bablock regression analyses for GDT-TS comparison between CONFOLD results of models generated using our program and other 3 methods on all available CASP12 targets.

Method Pairs	Regression	Parameters	Estimated Values	Lower bound *	Upper bound *
**DNCON2 vs. DeepConPred2**	Deming	Intercept	-0.0057	-0.0324	0.0209
		Slope	0.8962	0.7559	1.0364
	Passing-Bablock	Intercept	-0.0062	-0.0815	0.0383
		Slope	0.9290	0.7662	1.1372
**RaptorX-Contact vs. DeepConPred2**	Deming	Intercept	0.0124	-0.0518	0.0765
		Slope	1.1263	0.9088	1.3439
	Passing-Bablock	Intercept	0.0861	-0.0006	0.1662
		Slope	0.9021	0.7089	1.1099
**SPOT-Contact vs. DeepConPred2**	Deming	Intercept	0.0164	-0.0282	0.0610
		Slope	1.1424	0.9871	1.2976
	Passing-Bablock	Intercept	0.0485	-0.0163	0.1333
		Slope	1.0113	0.8307	1.1898

*with confidence of 95%.
